# Rapid Birth or Death of Centromeres on Fragmented Chromosomes in Maize

**DOI:** 10.1105/tpc.20.00389

**Published:** 2020-08-18

**Authors:** Yalin Liu, Handong Su, Jing Zhang, Lindan Shi, Yang Liu, Bing Zhang, Han Bai, Shuang Liang, Zhi Gao, James A. Birchler, Fangpu Han

**Affiliations:** a State Key Laboratory of Plant Cell and Chromosome Engineering, Institute of Genetics and Developmental Biology, Innovation Academy for Seed Design, Chinese Academy of Sciences, Beijing 100101, China; b University of Chinese Academy of Sciences, Beijing 100049, China; c Division of Biological Sciences, University of Missouri-Columbia, Columbia, Missouri 65211–7400

## Abstract

Comparative genomics has revealed common occurrences in karyotype evolution such as chromosomal end-to-end fusions and insertions of one chromosome into another near the centromere, as well as many cases of de novo centromeres that generate positional polymorphisms. However, how rearrangements such as dicentrics and acentrics persist without being destroyed or lost remains unclear. Here, we sought experimental evidence for the frequency and timeframe for inactivation and de novo formation of centromeres in maize (*Zea mays*). The pollen from plants with supernumerary B chromosomes was gamma-irradiated and then applied to normal maize silks of a line without B chromosomes. In ∼8,000 first-generation seedlings, we found many B–A translocations, centromere expansions, and ring chromosomes. We also found many dicentric chromosomes, but a fraction of these show only a single primary constriction, which suggests inactivation of one centromere. Chromosomal fragments were found without canonical centromere sequences, revealing de novo centromere formation over unique sequences; these were validated by immunolocalization with Thr133-phosphorylated histone H2A, a marker of active centromeres, and chromatin immunoprecipitation-sequencing with the CENH3 antibody. These results illustrate the regular occurrence of centromere birth and death after chromosomal rearrangement during a narrow window of one to potentially only a few cell cycles for the rearranged chromosomes to be recognized in this experimental regime.

## Abstract (precis)

Centromere death and birth can occur at experimentally recognizable frequencies and in a narrow developmental window, providing insight into centromere activity and karyotype evolution.

## INTRODUCTION

Two of the most common surviving chromosomal rearrangements in plant and animal karyotype evolution are end-to-end fusions and insertions of whole chromosomes into or near the centromeres of other chromosomes (Montefalcone et al., 1999; Lysak et al., 2006; Luo et al., 2009; Wade et al., 2009; Murat et al., 2010; Shang et al., 2010; Schubert and Lysak, 2011; Wang and Bennetzen, 2012; Wang et al., 2015; Hoang and Schubert, 2017; Tolomeo et al., 2017; Birchler and Han, 2018; Mandaková et al., 2020). These rearrangements, however, would predict the formation of dicentric chromosomes that would tend to destroy themselves via anaphase bridge formation and breakage (McClintock, 1931, 1941). Furthermore, the shifting of centromere position as polymorphisms within a species (Schneider et al. 2016; Zhao, et al. 2017) would also require the rapid formation of new centromere sites upon centromere deletion or inactivation on the progenitor chromosome. For such aberrations to survive, centromere inactivation and de novo formation must occur at a reasonable frequency and become established presumably over the time frame of one or at least a few cell cycles, although documenting the timing of the centromeric changes in state is difficult to establish. To examine this question of centromere birth and death, we sought an experimental system to determine if such events were sufficiently frequent to account for these evolutionary events, and to define the developmental timeframe over which they occur.

**Figure:**
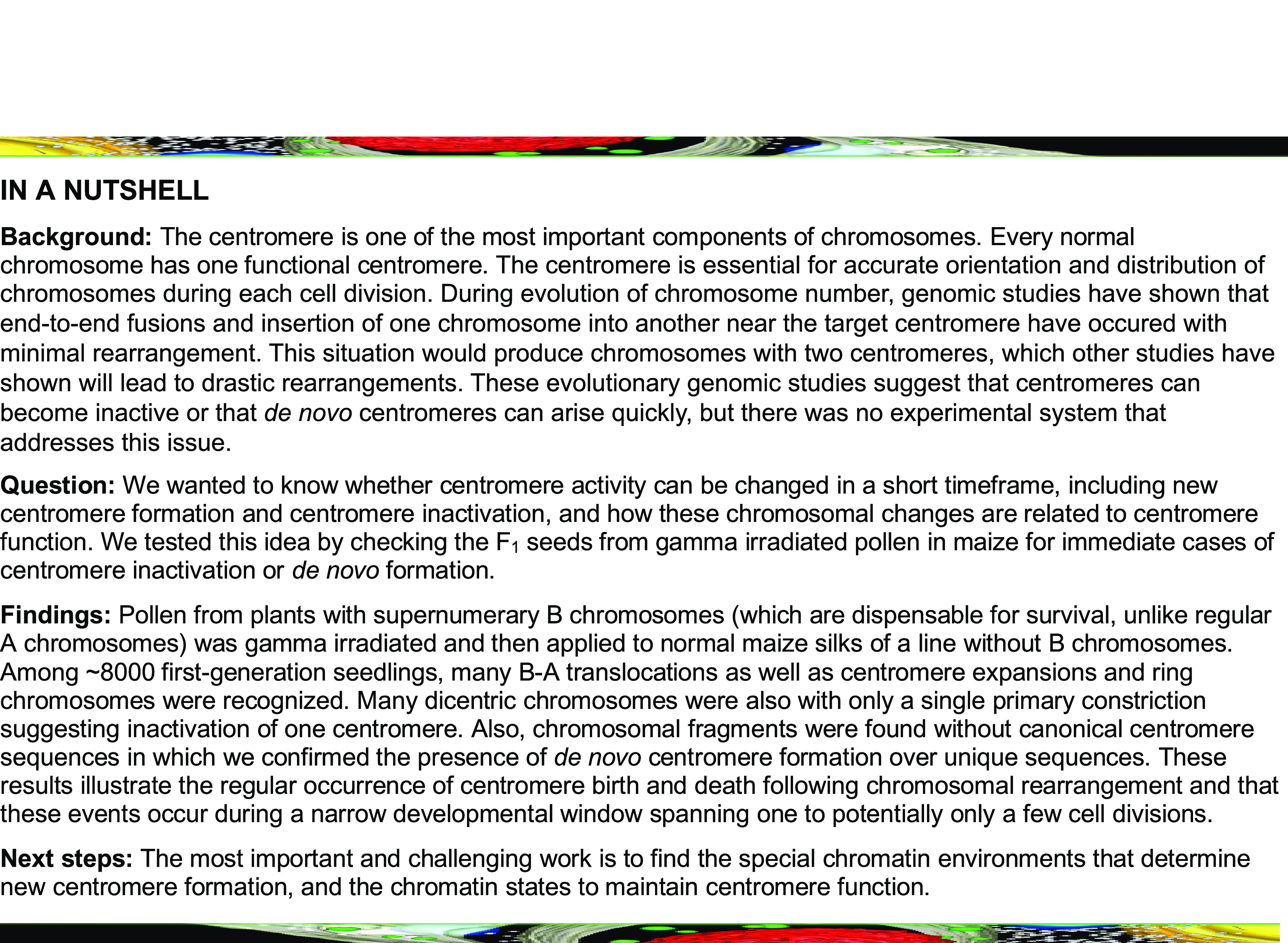


A case in the classical literature (Stadler and Roman, 1948) reported the recovery of a chromosomal fragment after pollen irradiation that lacked canonical centromere sequences but had established a de novo centromere (Fu et al., 2013). A case of a translocation recovered from exposure to an atomic bomb test produced an inactive centromere during formation of the aberration that has apparently remained inactive during propagation for >70 years (Gao et al., 2011; Albert et al., 2019). We employed an experimental design to test whether similar cases could validate the timeframe of establishment of the epigenetic states of centromeres that are implicated from the evolutionary genomics evidence. Toward this end, we conducted pollen irradiation on maize (*Zea mays*) material containing nonvital B chromosomes that have readily distinguishable cytological features. The immediate F1 was then screened for recognizable chromosomal changes via fluorescence in situ hybridization (FISH) on root tip metaphase spreads as an initial assay to detect cases of centromere formation or inactivation. Some cases were recovered in subsequent genetic crosses, which permitted molecular confirmation of the cytological determinations.

In maize, there are two types of centromeric specific repeats in the A centromeres, referred to as tandem repeat “CentC” and centromeric retrotransposon of maize (CRM; Ananiev et al., 1998; Kato et al., 2004; Birchler and Han, 2009). These sequences are shared with the B-chromosome centromere, which is also interspersed with a B-chromosome–specific repeat (Alfenito and Birchler, 1993; Kaszás and Birchler, 1998; Jin et al., 2005; Lamb et al. 2005).

The B chromosome also has a heterochromatic knob region composed of 180-bp repeats near the centromere (Lamb et al., 2005). The short arm of the B chromosome mainly contains B-repeat sequences and the long arm contains numerous sites of CentC homology that show no evidence of involvement in centromere function (Lamb et al., 2005). On the distal tip of the long arm of the B chromosome, there is a small region containing B-repeat and CentC but not CRM; the size of the B-repeat and CentC sites in the distal tip is much smaller than the core-B–centromeric region (Alfenito and Birchler, 1993; Lamb et al., 2005). Thus, the centromeric region, chromosomal arm, and distal chromosomal tip of the B chromosome can be recognized by the distributions of different sequence elements (Lamb et al., 2005), which facilitate the recognition of chromosomal rearrangements involving the B chromosome using a simple FISH probe cocktail.

Functional centromeres in plants are marked by a centromeric-specific H3 variant named “CENH3” (Talbert et al., 2002; Zhong et al., 2002). It is present at the site of all known functional centromeres and absent in all known cases of inactivated derivatives (Han et al., 2006, 2009, 2018; Gao et al., 2011). Also, Thr133-phosphorylated histone H2A is a biochemical marker of functional centromeres (Dong and Han, 2012).

Tassels from maize inbred line B73 with one or two B chromosomes (Supplemental Figure 1) were used for irradiation with x-rays from ^60^Co. The irradiated pollen was applied to the silks of inbred line B73 without B chromosomes. The use of a uniform B73 background facilitated chromosomal identification and subsequent molecular analyses using the B73 reference sequence (Jiao et al., 2017). The progeny seedlings were used for screening for chromosomal variants. Such variants were identified among ∼8,000 individuals and characterized with most attention given to centromere arrangement and function.

## RESULTS

### Variety of Chromosomal Aberrations Induced by Irradiation

**Figure 1.**
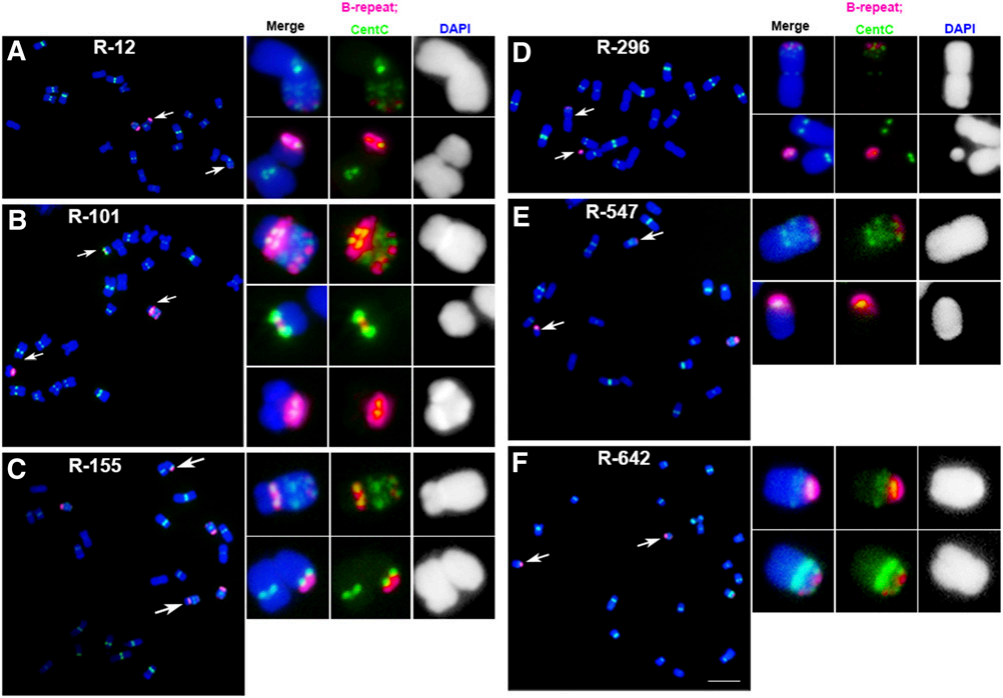
Examples of Reciprocal Translocations Observed. One of the most common chromosomal aberrations expected after irradiation is reciprocal translocations that exchange distal parts of nonhomologous chromosomes. Because the B chromosome contains CentC clusters at several sites along its length but the A-chromosomal sites of this satellite repeat are only found at centromeric regions, it is possible to detect reciprocal translocations between the B chromosome and A chromosomes. The B-chromosome centromere is unique in that it contains a specific centromere repeat for its identification. The following are examples of B–A translocations observed: **(A)** In event R-12, a reciprocal translocation occurred between the pericentromeric regions of the B chromosome and the short arm of an A chromosome, with the whole B-centromere region fused to the A-chromosomal arm. **(B)** In event R-101, the fragments from one B chromosome were translocated to three fragments from one A chromosome. **(C)** The centromeric regions of both B- and A chromosomes were apparently broken and fused several times. Reciprocal translocation between both the short arm of the B chromosome and the pericentromere region of an A chromosome was found in event R-155. **(D)** In event R-296, a reciprocal translocation placed the terminus of the B chromosome onto an A chromosome. **(E)** Reciprocal translocation between both the pericentromeric regions of B- and A chromosomes was found in event R-547. **(F)** Reciprocal translocation between the chromosomal arms of the B- and an A chromosome was found in event R-642. Scale bar = 10 μm. Magenta signals, B-repeat; green signals, CentC signals; blue, chromosomes counterstained with DAPI (but shown in grayscale in the insets for better visualization of the primary constrictions and knob heterochromatin).

Two-hundred and seventy-four chromosomal variants were identified after screening ∼8,000 seedlings by FISH (Table 1; Supplemental Data Set). Many variants are translocated, fragmented, or rearranged chromosomes (Table 1; Supplemental Data Set). Reciprocal translocation events were observed (Figure 1). For example, a reciprocal translocation occurred between the pericentromeric regions of the B chromosome and the short arm of an A chromosome, with the whole B-centromere region fused to the A-chromosomal arms in event R-12 (Figure 1A). Examples of other types of translocations are also illustrated (Supplemental Figures 2 and 3).

**Table 1.** Types of Recognizable Chromosomal Variants After Pollen Irradiation

Chromosomal variant	Number
A–B dicentrics	37
A–A dicentrics with two constrictions	46
B–B dicentrics	8
Multicentrics with multiple constrictions	3
A–A dicentrics with only one constriction	16
miniB	70
miniA	25
Ring chromosomes	6
A fragments lacking centromere sequences	8
B fragments lacking centromere sequences	1
Isochromosomes	2
Miscellaneous rearrangements	3
B–A translocations with no centromere variation	41
Deleted A	3
Deleted B	5

**Figure 2.**
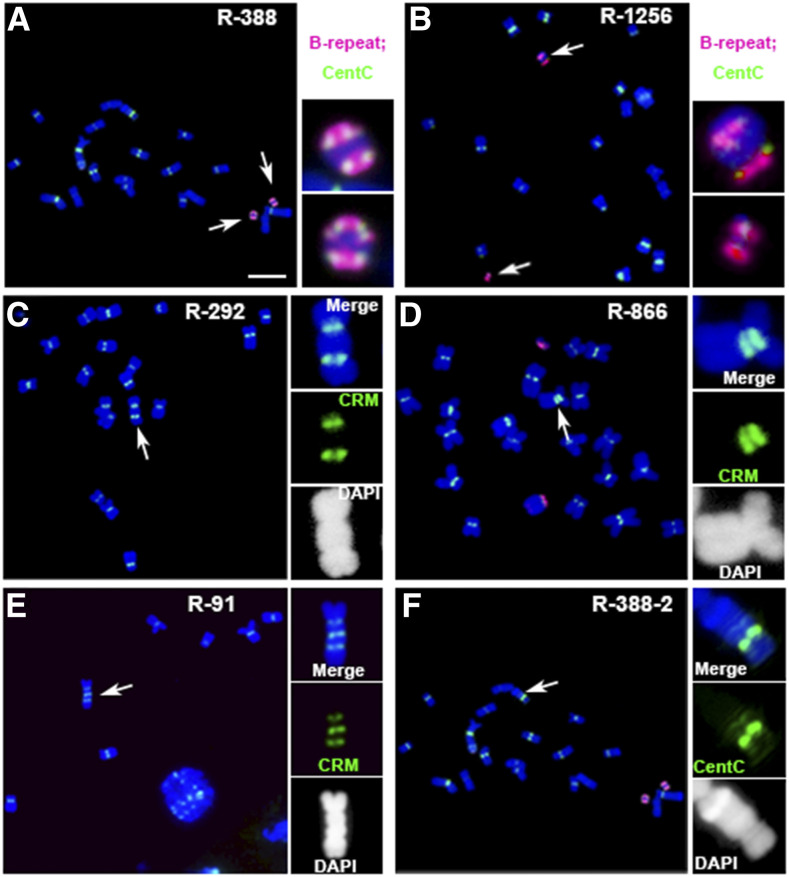
Examples of Multicentric Chromosomes. If broken chromosomes fuse so that the centromere proximal portions join, a multicentric chromosome is formed. Unless the centromeres are in close proximity to each other, they may proceed to different poles during anaphases and break the chromosome. Dicentric chromosomes with two B-repeat containing regions such as event R-388 **(A)** and R-1256 **(B)** were found. The following are examples of multicentric chromosomes observed: **(A)** In R-388, there are two similar dicentric chromosomes; both B-centromeric regions have strong B-repeat (magenta) and CentC signals (green). Scale bar = 10 μm. **(B)** In R-1256, a large dicentric chromosome and a small one were present, of which both the B-centromeric regions have B-repeat (magenta) and CentC signals (green). **(C)** The dicentric chromosome in event R-292 may become broken in the subsequent divisions or become stable by inactivating one centromere. **(D)** In event R-866, the two centromeric regions may be fused together to form a larger one, as the two sites are in close proximity. **(E)** and **(F)** Tricentric chromosome in event R-91 **(E)** and a chromosome with several CentC signals (green) containing regions in event R-388 **(F)** were found. These chromosomes are likely not stable, as multiple constrictions were present on the same chromosome. In the dicentric chromosomes, one centromere may become inactivated, or the two active centromeres may proceed to opposite poles during anaphases and cause breakage to produce monocentric derivatives. **(C)** to **(E)** Magenta signals, CRM; blue, chromosomes counterstained with DAPI (but shown in grayscale in the insets for better visualization of the primary constrictions and knob heterochromatin).

**Figure 3.**
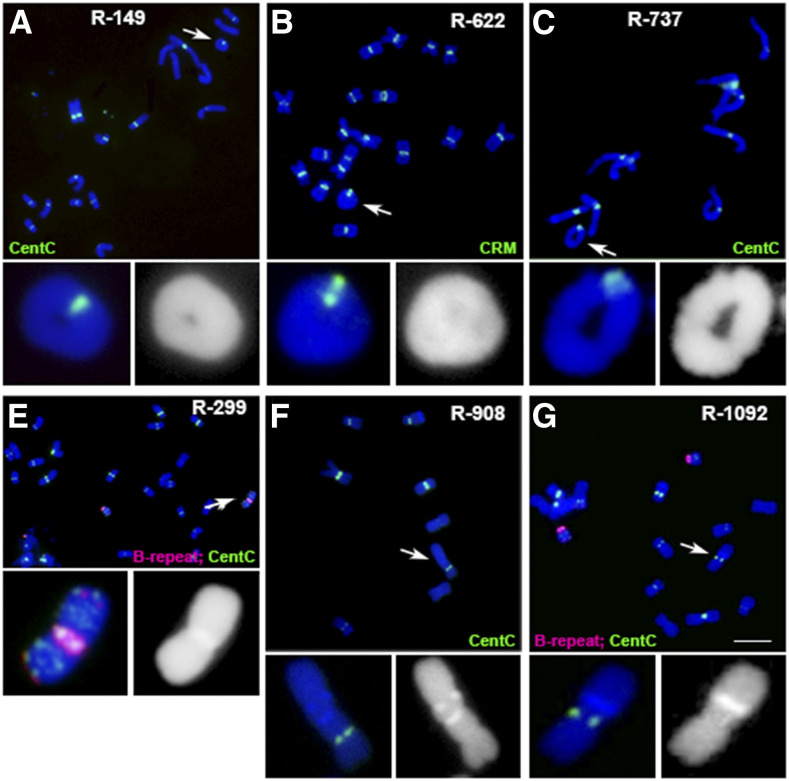
Rings and Other Rearrangements. When two breaks occur in a chromosome on opposite sides of a centromere and fusion includes the centromere, a ring chromosome results. The following are examples observed: **(A)** to **(C)** Different sizes of ring chromosomes were identified. These ring chromosomes are unlikely to be transmitted stably in most cases and break and rejoin during development (McClintock, 1932). Changes in the karyotype have been found in several A-chromosomes, according to the locations of knob heterochromatin regions on the chromosomes. In normal cells, knob regions are located at the end or toward the end of chromosomal arms. In **(A)** and **(C)** green signals, CentC. In **(B)**, green signals, CRM. **(D)** In event R-299, the iso-B chromosome may have been generated by fusion of the two broken centromere regions of two B chromosomes after pollen irradiation. **(E)** In event R-908, there is a long chromosomal arm with two knob regions positioned near the middle of the long arm, which might have resulted from fusion of two chromosomal fragments. Green signals, CentC. **(F)** In event R-1092, the long arm of one A chromosome may have an inversion to transfer the knob region near to the centromere. **(D)** and **(F)** Magenta and green signals, B-repeat and CentC, respectively. Blue, chromosomes counterstained with DAPI (but shown in grayscale in the insets for better visualization of the primary constrictions and knob heterochromatin). Scale bar = 10 μm.

Among the A-chromosome variants with A-centromere changes, there are fragments containing different sizes of A-centromeric regions, dicentric chromosomes with two primary constrictions, chromosomes with only one primary constriction but two sets of centromeric sequences, and A chromosomes containing three or more centromeric-sequence–containing regions (Figure 2; Table 1; Supplemental Figure 4; Supplemental Data Set). Centromeric fragments from A chromosomes were found that have different compositions and organizations of centromeric sequences that differ from the normal centromeres (Supplemental Figure 4). Different sizes of ring and other rearranged chromosomes with no detectable changes of centromeric repeats were also identified (Figure 3; Table 1; Supplemental Data Set). These examples illustrate the ability to recognize reciprocal translocations, multicentric chromosomes, minichromosomes, and other aberrations arising in the experiment.

### Inactive Centromeres on Translocation Chromosomes

Thirty-seven chromosomes transferred the core-B–centromeric region to the ends of A chromosomes; of these, dicentric chromosomes are the major type (Table 1; Supplemental Data Set). Terminal-B–chromosome centromeres that become attached to other chromosomes are difficult to classify with regard to activity because there is no flanking chromatin surrounding the B centromere to verify a primary constriction at the B centromere, which serves as a cytological criterion for activity. However, an example of an A–B dicentric was recovered and analyzed.

**Figure 4.**
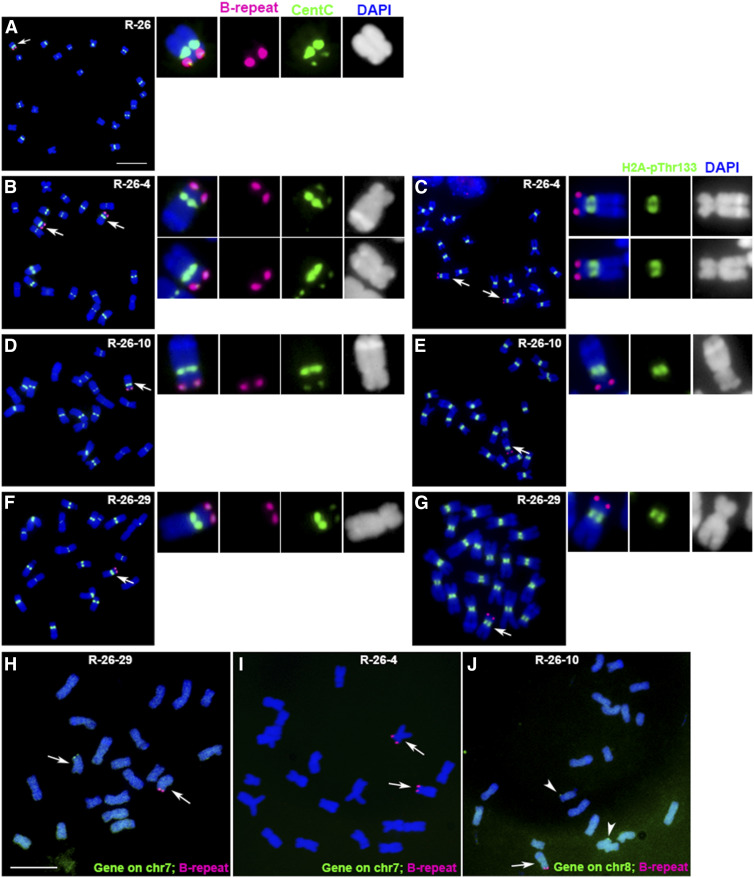
Confirmation of Centromere Inactivation in a Dicentric Chromosome. **(A)** In event R-26, the B centromere (magenta) was translocated to the chromosomal end of an A chromosome. **(B)** In the offspring, progeny R-26-4 has two dicentric chromosomes; one has a large knob region and the other has a small knob, suggesting that a translocation with another chromosome might have occurred in the opposite arm and recombined to join the different sized knobs with the adjunct portion of the B chromosome. **(C)**, **(E)**, and **(G)** All the B centromeres (magenta) in these dicentric chromosomes from event R-26 have no H2A-pThr133 signals (green), indicating that the B centromeres are inactive. The dicentric chromosomes in the progeny of event R-26 were probed via FISH for genes in the terminal regions of chr7 (Zm00001d018596, Chr7:290,154-295,438) and chr8 (Zm00001d008176, Chr8:327,598-335,271). **(D)** and **(F)** In other progeny individuals, there is a large knob in R-26-10 **(D)**, and a small knob in R-26-29 **(F)**. **(H)** In R-26-29, there was one dicentric chromosome and one chr7 signal (green) on the other chromosome. Scale bar = 10 μm. **(I)** In R-26-4, there were two dicentric chromosomes and no chr7 signals (green). **(J)** In R-26-10, there was one dicentric chromosome and two chr8 signals (green) on two other chromosomes. These results demonstrate that the dicentric chromosome in event R-26 is derived from chr7 but the most distal gene on the short arm of chr7 was deleted in the process. Blue, chromosomes counterstained with DAPI (but shown in grayscale in the insets for better visualization of the primary constrictions and knob heterochromatin).

In this example, the B centromere region was translocated to the end of an A chromosome in event R-26 (Figure 4A). While it was impractical (or indeed would be impossible for highly sterile events) to attempt to recover all observed aberrations in the next generation, this event was perpetuated in the offspring of a self-pollination. In one such progeny, R-26-4 has two dicentric chromosomes (Figure 4B). The dicentric chromosome has a large knob heterochromatin region in individual R-26-10 (Figure 4D), and has a small knob region in individual R-26-29 (Figure 4F), suggesting that a translocation with another chromosome likely occurred in the opposite arm, which then recombined in the F1 with the B centromere on the opposite terminus of the chromosome. The karyotype of inbred line B73 implicated chromosomes 7 or 8 in this aberration (Albert et al., 2010). The dicentric chromosomes in the progeny of event R-26 were examined using probes prepared from genes in the terminal regions of chr7 (Zm00001d018596, Chr7:290,154 to 295,438) and chr8 (Zm00001d008176, Chr8:327,598-335,271). In individual R-26-29, there was one dicentric chromosome and one chr7 signal on the other chromosome (Figure 4H). In individual R-26-4, there were two dicentric chromosomes and no chr7 signals (Figure 4I). In individual R-26-10, there was one dicentric chromosome and two chr8 signals on two other chromosomes (Figure 4J). These results show that the dicentric chromosome in event R-26 is derived from chr7, and that the very distal tip on the short arm of chr7 was lost.

Because this dicentric chromosome does not continually fracture across different cells and different generations, the activity of the centromeres was tested using immuno-fluorescence assays for H2A-pThr133, which is a biochemical marker of active centromeres (Dong and Han, 2012). All the B centromeres in these dicentric chromosomes from event R-26 have no H2A-pThr133 signal, indicating that the B centromeres are inactive (Figures 4C, 4E, and 4G). Event R-26 serves as an example of an inactive centromere on a chromosome with a single primary constriction that was analyzed for biochemical features of activity. Others with only a single primary constriction are also likely cases of chromosomes with an inactive centromere (Table 1; Supplemental Data Set), which previous studies have indicated can occur regularly (Han et al., 2006).

Twenty-six A–A chromosomes with two sets of centromeric sequences were found that possess only a single primary constriction (Table 1; Supplemental Data Set). These can be readily distinguished from chromosomes with multiple primary constrictions as revealed in grayscale for the 4′,6-diamidino-2-phenylindole (DAPI) staining (Figure 2). Based on the criterion of a single primary constriction in these chromosomes, they likely represent cases in which one set of centromere sequences is inactivated. A previously analyzed chromosome, Translocations 1 to 5 (8,041), possesses two strong canonical centromere sequence sites but only a single position of CENH3 accumulation associated with the sole primary constriction (Gao et al., 2011; Albert et al., 2019). In hybrids between common wheat (*Triticum aestivum*) and *Thinopyrum elongatum*, chromosomes with two or three sets of centromeric sequences were observed, but only one set was associated with CENH3 immunostaining, and it corresponded to the only constriction (Guo et al., 2016). A tricentric chromosome in wheat with multiple sites of CENH3 exhibits cytological constrictions associated with the CENH3 positions (Zhang et al., 2010). These examples illustrate that the cytological feature of a single constriction on a chromosome with two or more sets of centromeric sequences is a sign of centromere inactivity, and that the lack of a primary constriction at the site of centromeric sequences is an indicator of inactivity.

### Fragments Lacking Centromere Sequences

Among the A-chromosomal variants, there were fragments with no detectable centromeric-specific sequences (Table 1; Supplemental Data Set). In event R-109, a small A-chromosomal fragment has no detectable centromeric repeats from probings with the B-repeat, CentC, and CRM, suggesting other genomic DNA may be involved in centromere function (Supplemental Figures 5A and 5B). De novo centromeres could be formed on such fragments.

**Figure 5.**
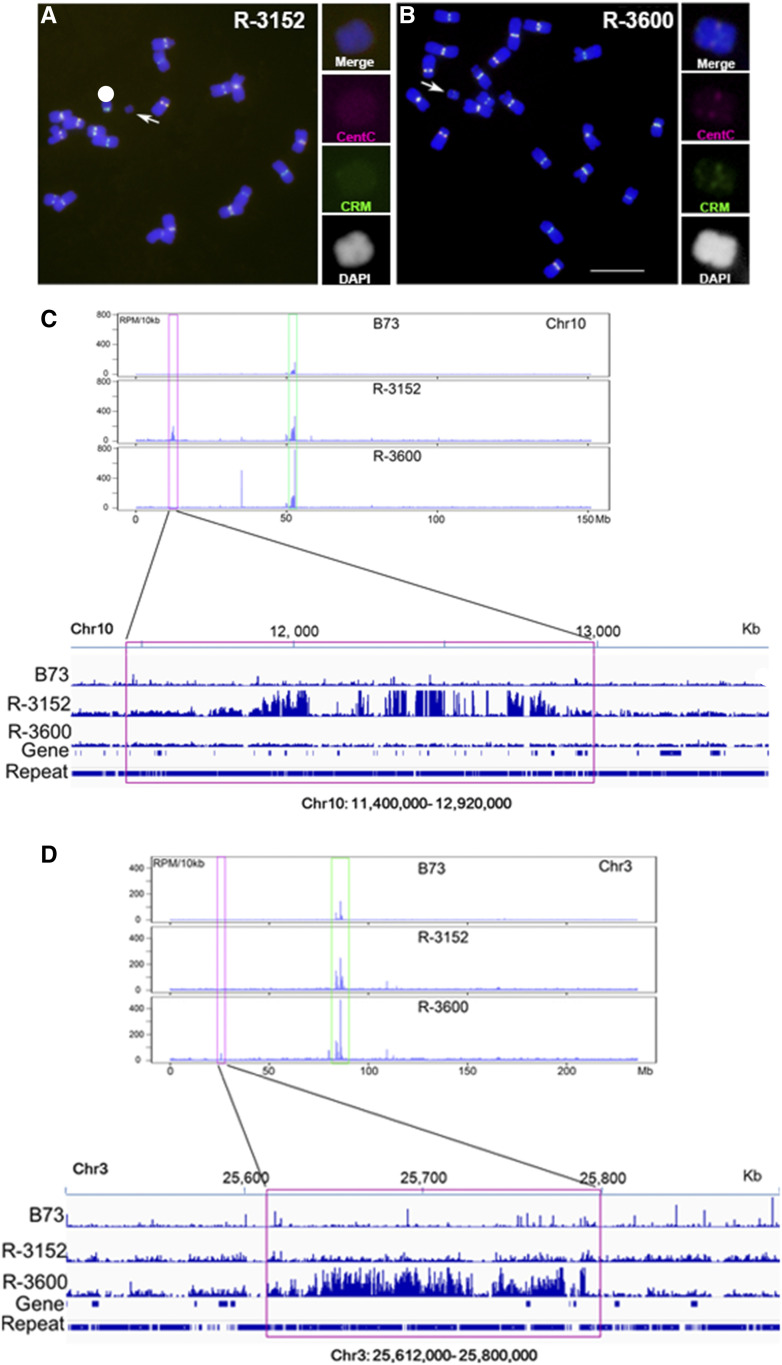
Confirmation of De Novo Centromere Formation on Acentric Fragments. **(A)** Two A-chromosomal fragments with deleted or reduced centromeric sequences were used to determine the DNA sequences associated with centromere function. In event R-3152, there are no FISH-detectable CentC (magenta) and CRM (green) signals on the small chromosomal fragment. **(B)** In R-3600, there are very weak CentC (magenta) and CRM (green) signals on the small chromosomal fragment, suggesting deletion of most of a centromere. Scale bar = 10 μm. **(C)** Anti-CENH3 ChIP-seq was conducted to check the functional centromeric regions marked by CENH3 nucleosomes. A 1,520-kb genomic region from the short arm of chromosome 10 (chr10: 11,400,000 to 12,920,000) was found to be enriched with reads from the anti-CENH3 ChIP-seq data. This region is ∼40 Mb removed from the native centromere 10. **(D)** Anti-CENH3 ChIP-seq was conducted on this material. A 188-kb genomic region was found from the short arm of chromosome 3 (chr3: 25,612,000 to 25,800,000) with enriched reads from the anti-CENH3 ChIP-seq data. In chromosome 3, the CentC- and CRM-enriched native centromere region is >50 Mb from the 188-kb CENH3 binding region. Blue, chromosomes counterstained with DAPI (but is shown in grayscale in the insets for better visualization of the primary constrictions and knob heterochromatin).

Two A-chromosomal fragments with deleted or reduced centromeric sequences were recovered and used to determine the DNA sequences associated with centromere function. In event R-3152, there are no FISH-detectable CentC or CRM signals on the small chromosomal fragment (Figure 5A). Anti-CENH3 chromatin immunoprecipitation sequencing (ChIP-seq) was conducted to check the functional centromeric regions marked by CENH3 nucleosomes. A 1,520-kb genomic region from the short arm of chromosome 10 (chr10: 11,400,000 to 12,920,000) was enriched with reads from the anti-CENH3 ChIP-seq data. This region is ∼40 Mb removed from the native centromere 10 (Figure 5C). These results revealed that CENH3 nucleosomes are localized in this region to form a functional centromere on the fragment of event R-3152.

In event R-3600, there are very weak CentC and CRM signals on the small chromosomal fragment, which might have resulted from deletion of most of a centromere (Figure 5B). Anti-CENH3 ChIP-seq was conducted on this material. A 188-kb genomic region from the short arm of chromosome 3 (chr3: 25,612,000 to 25,800,000) was found to be enriched with reads from the anti-CENH3 ChIP-seq data (Figure 5D). The sequences from this region are involved in the functional centromeric region of the fragment in R-3600. In chromosome 3, the CentC- and CRM-enriched native centromere region is >50 Mb removed from the 188-kb CENH3 binding region.

## DISCUSSION

**Figure 6.**
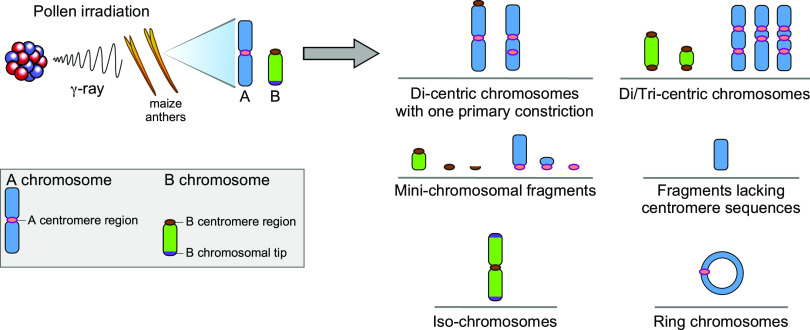
Summary of Chromosomal Aberrations Observed. The types of chromosomal aberrations recognized are shown. Key to the chromosomal features is at the right. After pollen irradiation and screening of the immediate F1 seedlings, multiple types of aberrations were found with special emphasis on centromeric changes (Table 1). Dicentric and multicentric chromosomes were observed as well as acentric fragments. Dicentric chromosomes with a single primary constriction are candidates for centromere inactivation. Acentric fragments that are inherited are candidate chromosomes with de novo centromeres. Additional chromosomal types included various sizes of minichromosomal fragments with centromeres of various sizes, an isochromosome, and ring chromosomes.

Here, we sought to establish the minimal developmental timeframe over which centromere formation or inactivation can occur. Dicentric chromosomes will typically break and drastically rearrange, and acentric fragments will be lost (McClintock 1931, 1941), but evolutionary genomics has shown their apparent repeated occurrence in karyotype evolution. Further, studies have shown the prevalence of centromere site polymorphism (Schneider et al., 2016), again raising the question of how this is possible. Toward this end, the experimental setup of irradiating mature pollen and screening the immediate F1 avoids the possibility of selection against any chromosomal changes, with the possible exception of extreme aneuploidy that might be lethal to early embryos. As anticipated, numerous translocations, minichromosomes, and fragments were found (Figure 6; Table; Supplemental Data Set). Of special interest were cases of rearrangements that contain multiple sequences typical of centromeres or those lacking such sequences. Previous studies of chromosomes with two sets of centromeric sequences but that are stable achieve that state via inactivation of one set of sequences to exhibit a single primary constriction typical of a functional centromere (Han et al., 2006; Gao et al., 2011). Fragments lacking canonical sequences can acquire de novo centromeres (Fu et al., 2013; Zhang et al., 2013; Liu et al., 2015; Su et al., 2016).

Mature pollen grains of maize contain two sperm, one of which fertilizes the egg to produce the zygote of the next generation. For any inactivation or de novo formation of centromeres to be recognized in multiple examined cells in the root tip preparations, these changes must occur during the first few cell divisions of development so as to be represented among most cells of the seedling. Fractured chromosomes in the sperm must be delivered to the egg at fertilization. The first division of the zygote does not occur until ∼10 to 12 h after fertilization (Kiesselbach, 1949; Sheridan and Clark, 1994), although the exact timing is likely subject to environmental conditions and genetic background. For those cases that were observed in the root tip spreads and recovered in the next generation from a self-cross, the change in the centromere function must occur during the early cell cycles of development after fertilization for them to be of uniform structure in multiple root tip cells and also inherited.

Numerous examples were found in which there was an A–B or an A–A chromosome with two sets of centromeric sequences that appeared to be stable by the criteria of having the same structure in the multiple cells examined and a single primary constriction. An example salvaged in the subsequent generation was used to biochemically confirm that only a single centromere possessed the molecular attributes of activity (Figure 4). Centromere inactivation on single-centromere chromosomes results in eventual chromosomal loss, whereas dicentric chromosomes retain a functional centromere after inactivation and can therefore be recovered.

It is not possible to determine if the frequency of centromere inactivation observed here occurs under normal circumstances or if it is accentuated by the dicentric state. Inactivation in normal chromosomes would result in chromosomal loss and likely selection against the affected cell. In contrast, when dicentrics are intentionally generated with large and small centromeres via recombination in special chromosomes, descendants of such chromosomes can be recovered with inactivated small centromeres (Han, et al., 2009, 2018). A related situation in *T. aestivum* has been documented (Zhang et al., 2010).

At a lower frequency than inactivation, chromosomal fragments were recognized that lacked detectable normal centromere sequences (Table 1). Two examples were recovered in the subsequent generation. ChIP-seq analysis using antibodies against CENH3 validated that de novo centromeres had arisen on them. One involved a portion of chromosome 10 and another a portion of chromosome 3. The de novo centromere on chromosome 10 lies very close to a silenced ancestral centromeric site on the short arm of chromosome 10 (Wang and Bennetzen, 2012; Schneider et al., 2016), although no detectable canonical centromere sequences are present at this position. The de novo centromere on chromosome 3 lies in the middle of a large and non-centromeric region (Schneider et al., 2016). A minor representation of centromere sequences is found on this chromosome, suggesting a complex origin with novel sequences becoming involved with centromere function. De novo centromere formation must have been established shortly after fertilization, and then was perpetuated to be observed in the examined cells in the root tip and subsequently inherited.

It is also not possible to determine whether the frequency of de novo centromere formation reflects the rate of such formation within chromosome arms under normal circumstances. De novo formation in a normal chromosome would produce two active centromeres, which would fracture the chromosome or erasure might occur of any smaller de novo centromeres in analogy to the cases described (Han et al., 2009, 2018; Zhang et al., 2010). An acentric chromosomal fragment might foster their generation and there would be no competition occurring. The recovery of fragments lacking canonical centromeric sequences and the validation of de novo formation illustrates that if a normal chromosome has its centromere deleted or inactivated, de novo centromere formation is sufficiently common to support the implications from comparative genomics of their involvement in karyotype evolution (Schneider et al., 2016).

Lewis Stadler described a phenomenon referred to as “deficiency recovery” (Stadler, 1930), which Barbara McClintock attempted to study on the chromosomal level (Birchler and Han, 2018). After irradiation of pollen with dominant genetic markers that was applied to silks of a recessive tester, the dominant markers in some individuals in the resulting plants were missing the linked paternal alleles, but occasionally they would reappear in later developmental sectors. These observations were made long before the genetic material was known and have since fallen into obscurity. Based on our results, a potential explanation for deficiency recovery might be that a dissociated acentric chromosomal fragment would acquire a de novo centromere and then become perpetuated in a sector of the resulting plant together with the corresponding deficiency. If the broken-off fragment is passively included into only one of the two daughter cells at each of the first few divisions of development and then acquires a de novo centromere, it could then be partitioned into both daughter cells in the subsequent divisions, forming a developmental sector with the dominant marker on a background of the recessive. McClintock (1938) found that acentric fragments released from an inversion heterozygote during meiosis can persist through subsequent divisions at random into the microspores and be visualized at the first microspore mitosis. Her finding illustrates that acentric fragments can be perpetuated into some of the daughter cells before being lost. The short timeframe of only one or a few cell cycles in which de novo centromeres can become established in early development as shown here can provide an explanation for deficiency recovery. This connection is potentially a further indication of the frequent and rapid formation of de novo centromeres.

Examples of chromosomal end-to-end fusions have been documented from evolutionary genomics and experimental circumstances (Montefalcone et al., 1999; Luo et al., 2009; Lysak et al., 2006; Wade et al., 2009; Murat et al., 2010; Shang et al., 2010; Schubert and Lysak, 2011; Wang and Bennetzen, 2012; Wang et al., 2015; Hoang and Schubert, 2017; Tolomeo et al., 2017; Birchler and Han, 2018; Mandaková et al., 2020). In this experiment, no cases of the attachment of a chromosomal fragment to the very terminus of another chromosome could be confirmed (Figure 4; Supplemental Figure 2). Such events might occur with the stochastic failure of telomere capping, with the chromosome terminus acting as a double-strand break and join, with other double-strand breaks produced in the cell as occurs with end-to-end fusion in telomerase mutants (Riha et al., 2001). Many nonreciprocal translocations were observed, but it was not possible to discern whether a broken chromosome attaches to the very end of the chromosome without removal of any genes. In example cases (Figure 4; Supplemental Figure 2), the aberration was confirmed to have lost the tip of the chromosome. However, we cannot conclude that attachments to telomeres fail to occur in other circumstances, as have been suggested in the literature (Birchler and Han, 2018).

Deletions, translocations, inversions, and multicentric chromosomes will usually be selected against due to the partial sterility that they would produce in individuals in a natural population (Burnham, 1962). Nevertheless, the involvement of these types of aberrations in karyotype evolution has been studied extensively over many decades. In recent years, comparative genomics has implicated centromere inactivation and de novo formation (Birchler and Han, 2018), which classically were not appreciated in genome evolution such that they must be relatively common with the altered functional states established rapidly and subsequently perpetuated. In the experimental system reported here, we have shown that to be the case.

## METHODS

### Plant Materials

The *Zea mays* B73+B and B73 inbred lines were screened by FISH using probes for CentC and CRM (Ananiev et al., 1998), knob heterochromatin (Peacock et al., 1981), and B-repeat (Alfenito and Birchler, 1993). The B73 and B73+B or B73+2B plants were grown at the genetic farm of the Institute of Genetics and Developmental Biology, Chinese Academy of Sciences. When the anthers on the tassel were mature, the whole tassels of B73+B or B73+2B plants were collected for irradiation with ^60^Co. The radiation dose was 18 Gray. After irradiation, pollen was collected and then applied to the silks of B73. The harvested seeds were germinated for FISH screening on the resulting seedlings. (The materials [seeds] are available from F.P.H. or J.A.B.)

### DNA Probe and FISH

For mitotic analysis, plasmids containing CentC, CRM, knob, and B-repeat were labeled with Alexa Fluor-488-5-dUTP or TEXAS RED-5-dCTP, by nick translation as described by Gao et al. (2011). Plasmids containing the DNA sequences of Zm00001d039211, Zm00001d018596, and Zm00001d008176 were labeled with Alexa Fluor-488-5-dUTP by nick translation. The primers used to amplify these genes from B73 genomic DNA are listed in Supplemental Table. Images were taken with confocal microscopy (Cell Observer SD; Zeiss) and processed with the software Photoshop CS 3.0 (Adobe). Interpretations of mitotic figures underwent an iterative process until consensus was reached among all authors.

### Immunolocalization in Mitotic Cells

Immunolocalization for mitosis was performed as described by Han et al. (2009). The maize anti-CENH3 antibody (Fu et al., 2013) and the phosphorylated H2A antibody (Dong and Han, 2012) were purchased from GL Biochem. The images were taken as described above.

### ChIP and ChIP-Seq

ChIP was performed as described by Nagaki et al. (2003). About 20 g of young leaves was used for ChIP. The enriched DNA samples were sequenced using the Hiseq2000 platform (Illumina) to generate pair-ended 100-bp sequence reads.

### Analysis of ChIP-Seq Data

About 300 to 400 megabases of ChIP-seq paired-end reads were mapped to Zea_mays.AGPv4 (Jiao et al., 2017) using the software BWA, as described by Li and Durbin (2009). Only uniquely mapping reads were chosen for further analysis. Then the abundance of reads was calculated by reads per million values with 10-kb windows sliding along the genomic regions of interest. Figures were produced with R scripts. The anti-CENH3 ChIP-seq data are in the Gene Expression Omnibus database under number GSE124242.

### Accession Numbers

The anti-CENH3 ChIP-seq data are in the Gene Expression Omnibus database under number GSE124242. The sequences used for FISH can be found under accession numbers Zm00001d039211 (NP_001278731), Zm00001d018596 (XP_008651903) and Zm00001d008176 (XP_008655451).

### Supplemental Data

**Supplemental Figure 1.** Examples of other types of translocations.
**Supplemental Figure 2.** Analysis of a terminal translocation.
**Supplemental Figure 3.** Examples of altered centromere structure.
**Supplemental Figure 4.** Fragment lacking detectable centromere sequences.
**Supplemental Table.** Primers used for amplifying FISH probes.
**Supplemental Data Set.** Recognizable chromosomal variants in each line after pollen irradiation.

## DIVE Curated Terms

The following phenotypic, genotypic, and functional terms are of significance to the work described in this paper:

CENH3 Gramene: NM_001112050
CENH3 Araport: NM_001112050

dapi CHEBI: CHEBI:51231
